# Institutional Notes and News

**Published:** 1910-11-12

**Authors:** 


### Institutional Notes and News.

**Essex and the County Hospital.**

The small attendance which reached the Moot Hall, Colchester, on November 2, at a meeting held there on behalf of the Essex County Hospital, had an experience which it will never forget. We refer to the candid philippic delivered by Sir Henry Burdett, who had been asked as principal speaker. The Mayor of Colchester, Councillor E. Alec Blaxhill, opened the proceedings by lamenting the smallness of the audience, and reminded it that his connection with the Colchester Hospital had lasted eleven years, and that he was now the senior member of the committee. He made the audience laugh by referring to the hospital's accumulated debt of £3,500, about which, he said, the public, like the committee, had grown very tired of hearing. He added that he was determined to leave the hospital better off at the end of his mayoralty than it was at the beginning. The three things needed for the hospital were the public sympathy, their personal service, and their money. "The hospital," concluded the Mayor, "has from time to time been the subject of a considerable amount of criticism." It was not the hospital at Colchester, however, but Colchester itself, which came under the rod of Sir Henry's irony that night : "Colchester," he said, having paid three visits to the town on the hospital's behalf at different periods, "struck chill on hospital subjects." One might imagine that sickness was unknown there, that sympathy was never felt there for the poor wayfaring man who fell out by the way through no fault of his own. Sir Henry then drew a comparison between Colchester and Chelmsford, reminding his audience of the response and enthusiasm that had been aroused in Chelmsford for a similar cause. Colchester, he supposed, had no pride of citizenship, and cared for nothing but to be remembered by the regularity of its oyster feasts. But he would remind them that besides the organ which agreed with the oyster, there were two others higher in the scale, the head and the heart, which agreed with more important things. To-day, however, these seemed to be atrophied; so obese, so lazy, had the people grown that instead of taking part in public affairs they left everything to the newspapers, and relied on them for their enthusiasm, their energy, and their education. Sometimes the Press did succeed in stirring up in its readers a sense of the value of these things, and that he was always grateful for. All the people learned nowadays was mainly from the newspapers, and he hoped the newspapers in Colchester would bring home to its inhabitants the claims of the hospital. The hospital never had been properly supported; in 1896 he remembered being called in for advice, and he came again now to find out what was wrong with its supporters. The public ought to be grateful to the committee for their economical management of the hospital; but, instead of showing that it was so, the receipts of the Saturday and Sunday Funds were decreasing steadily. The only things to be done were to turn to the working men, who were always loyal to the hospitals; to press ladies into the service of Mrs. Egerton Green's linen guild; and to keep an account of the numbers of cases received from every town and village, showing side by side with that whether the support received was adequate to meet the cost of those cases. If it were not, that town or village should be asked the reason why. Sir Henry Burdett further suggested that a hospital association should be formed, each of the members of which should collect 5s. a year, which would result, he believed, in increasing the income by £1,000. Sir Henry concluded by a promise to help them in any way he could, and was enthusiastically cheered at the close. A resolution in support of the hospital was then carried, Mr. Benham, the Deputy-Mayor, wittily remarking that the empty chairs were a forcible argument in its favour. The audience, however, was by this time thoroughly stirred, and its measure was given accurately by Alderman Asher Prior, who said "If we are not aroused by this indictment, the most scathing even against any criminal I have ever heard in a long professional career, to carry out the suggested schemes, I shall feel that we are accessories after the fact." (Loud applause.) Thus ended a meeting which no one present is likely to forget, and which everyone present will feel proud of having attended.

**West Kent General Hospital.**

A Meeting was held recently at the Town Hall, Maidstone, to consider what form the memorial to his late Majesty King Edward should take. The memorial proposed is a scheme to extend the West Kent General Hospital and to increase the accommodation from sixty-seven to a hundred beds, with a suggestion that such additional building, or beds, should be called the "King Edward Wing" or the "Edward Beds." The scheme for extension is based upon the fact that the hospital receives patients from no fewer than ninety parishes—that is to say, it comprises a population of 82,000 persons. A sum of £20,000 is necessary for the building and endowment. Several substantial sums were promised at the meeting. There can be little anxiety concerning the success of the scheme if the above facts are brought forward prominently and enthusiastically enough. The Southern Counties will accept cheerfully the task of proving that agricultural and hopping England is quite as ready as the industrial North to honour the memory of the late King by associating it, no less closely after than before his death, with our voluntary hospitals.

**Country Hospital for Children, Liverpool.**

It was a revelation to those ladies and gentlemen who accompanied the Lord Mayor of Liverpool on the invitation of the Institution's Committee, to find what a fine building the Royal Liverpool Country Hospital for Children at Heswell is. Scarcely two years old, though the idea at the back of it was originated as far back as 1899, this hospital was the first to organise active medical and surgical treatment of sick children in the country itself; and thereby gave to the now hackneyed term, open-air treatment, a breadth of view and significance which its absurd restriction to sanatorium life had actually obscured, in the public mind at any rate. Liverpool is one of those cities which likes to do thoroughly what it undertakes to do at all; and so the above hospital was designed on a scale to accommodate some two hundred children. The administration block has been completed, so have two large wards, while two more are now actually being built; when finished and occupied the number of available beds will have risen to one hundred and thirty. As it might be thought that where fresh air was the first consideration buildings of any kind would be superfluous, we hasten to add that the finished wards have backs and two sides only, the fronts being as much open to air and sunlight as any room, on the stage, is open to the audience. In the light of such originality of conception the debt of £6,000 is a return unworthy of Liverpool, and, we are sure, soon to be paid.

